# Focal overexpression of CEACAM6 contributes to enhanced tumourigenesis in head and neck cancer *via* suppression of apoptosis

**DOI:** 10.1186/1476-4598-11-74

**Published:** 2012-09-28

**Authors:** Sarina Cameron, Lilia Merida de Long, Mehlika Hazar-Rethinam, Eleni Topkas, Liliana Endo-Munoz, Andrew Cumming, Orla Gannon, Alexander Guminski, Nicholas Saunders

**Affiliations:** 1 University of Queensland Diamantina Institute, Epithelial Pathobiology Group, Princess Alexandra Hospital, Queensland, Australia; 2 University of Queensland Diamantina Institute, Princess Alexandra Hospital, Ipswich Road Woolloongabba, Queensland, 4102, Australia

**Keywords:** CEACAM6, HNSCC, Tumour initiation, Cleaved Caspase 3

## Abstract

**Background:**

Overexpression of CEACAM6 has been reported for a number of malignancies. However, the mechanism of how CEACAM6 contributes to cancer formation and its role in head and neck squamous cell carcinoma (HNSCC) remains unclear. Therefore, we examined the role of CEACAM6 in head and neck squamous cell carcinoma (HNSCC).

**Methods:**

CEACAM6 expression was examined in normal squamous epithelia as well as a number of patient HNSCC samples and tumours derived from HNSCC cell lines injected into NOD/SCID mice. CEACAM6 expression was manipulated in HNSCC cell lines by shRNA-mediated CEACAM6 knockdown or virally-delivered overexpression of CEACAM6. The role of CEACAM6 in tumour growth and chemotherapeutic sensitivity was then assessed *in vivo* and *in vitro* respectively.

**Results:**

CEACAM6 expression was significantly increased in highly tumourigenic HNSCC cell lines when compared to poorly tumourigenic HNSCC cell lines. Moreover, HNSCC patient tumours demonstrated focal expression of CEACAM6. Functional investigation of CEACAM6, involving over-expression and knock down studies, demonstrated that CEACAM6 over-expression could enhance tumour initiating activity and tumour growth *via* activation of AKT and suppression of caspase-3 mediated cell death.

**Conclusion:**

We report that CEACAM6 is focally overexpressed in a large fraction of human HNSCCs *in situ.* We also show that over-expression of CEACAM6 increases tumour growth and tumour initiating activity by suppressing PI3K/AKT-dependent apoptosis of HNSCC in a xenotransplant model of HNSCC. Finally, our studies indicate that foci of CEACAM6 expressing cells are selectively ablated by treatment of xenotransplant tumours with pharmacological inhibitors of PI3K/AKT *in vivo*.

## Introduction

CEACAM6 is a member of the cacinoembryonic antigen (CEA) family of immunoglobulin glycoprotein cell adhesion molecules (CAM) comprising at least 12 CEACAM members [1]. CEACAMs are a diverse group of proteins which play major roles in cell-cell and cell-ECM adhesion and have been implicated in the control of cell proliferation, angiogenesis and tissue remodelling [1]. More recently, CEACAMs have also been implicated in mediating tissue responses to pathogens [1]. CEACAM6 is expressed at low levels in normal epithelial, endothelial and hematopoetic cells including granulocytes, T-cells and NK cells [2-4]. In contrast, CEACAMs are up-regulated in many epithelial malignancies including pancreatic, colorectal and breast cancers [5,6]. The expression of CEACAM6 also correlates with the metastatic potential of some epithelial malignancies, suggesting that the altered expression of CEACAM6 may contribute to tumour progression [7]. However, a definitive role for CEACAMs in tumourigenesis has not been formally proved. For example, CEACAM6 appears to affect the release of cytochrome-c from the mitochondria in response to cell detachment leading to the inhibition of caspase activation and hence, suppression of caspase induced apoptosis or anoikis in pancreatic cancer cells [8,9]. These apoptotic-suppressive effects have been shown to be AKT-dependent in pancreatic cancer cells [9]. Moreover, transgenic mice which overexpress members of the CEA family display colonic dysplasia [10]. In contrast, CEACAM6 up-regulation is associated with an increase in apoptosis in acute lymphoblastic leukaemia (ALL), indicating that the apoptosis-modulating effects of CEACAM6 may be tumour-type-specific [4].

A recent transcriptomic profiling study comparing highly tumourigenic clonal variants of an established head and neck cancer squamous cell carcinoma (HNSCC) cell line with poorly tumourigenic clonal variants, identified a strong association between CEACAM6 expression and tumourigenic potential [11]. Since an association between HNSCC and CEACAM6 expression has not been previously reported we now examine whether the over-expression of CEACAM6 is also present in human HNSCC samples.

## Materials and methods

### Cell culture and patient tumours

All HNSCC cell lines were obtained from the ATCC and cultured as per ATCC recommendations (Sydney, NSW, Australia). Patient tumour samples were all confirmed as invasive squamous cell carcinoma (SCC) by a staff Pathologist (Princess Alexandra Hospital). Overall we examined 4 tongue SCC, 3 lip SCC and normal mucosae from all these patients. Normal human epidermal keratinocytes (HEKs) were isolated and cultured from neonatal foreskin samples following circumcision as described [12,13]. Patient consent and approval by the Princess Alexandra Hospital Human Ethics Committee was obtained for all samples collected.

### Reverse transcriptase and real-time PCR (rt PCR)

Total RNA was isolated from cell lines with the addition of trizol (Invitrogen, Melbourne, VIC, Australia) as per manufacturer’s instructions. Quantification and reverse transcriptase reaction was performed as previously described [12]. The rtPCR CEACAM6 forward primer 5’ GACAGTTCCATGTATACCCG 3’ and the reverse primer 5’ACAGCATCCTTGTCCTCC 3’, were obtained from Sigma-Aldrich (Sigma-Aldrich, Sydney, NSW, Australia). The rtPCR reaction solutions were prepared and performed as per manufacturer’s instructions (Promega, Sydney, NSW, Australia). RtPCR reactions were performed as previously described [14].

### Western blot analysis

Total cellular protein was isolated using RIPA buffer and quantified as previously described [15]. Up to 20μg of protein was loaded onto a 10% SDS-PAGE, transferred onto PVDF membrane and probed as previously described [15]. A 1/1000 dilution of anti-CEACAM6 antibody (Abcam, Sapphire Bioscience, Sydney, NSW, Australia), 1/1000 dilution of of anti-AKT or anti-phospho S473AKT [13] and a 1/1500 dilution of the secondary anti-mouse Horse Radish Peroxidase (HRP) (GE Healthcare, Sydney, NSW, Australia) antibody was used to detect protein using chemiluminescence as per manufacturer’s instructions (Pierce, Rockford, IL, USA). Western blots were stripped as per manufacturers instruction (Thermo Scientific, Rockford, Il, USA) to re-probe with a 1/1000 dilution of β actin antibody (Sigma-Aldrich) and a 1:2000 dilution of the anti-Rabbit HRP (GE Healthcare) secondary antibody.

### Cell proliferation and death assays in vitro

Bromo-deoxy uridine (BrdU) incorporation was used to estimate proliferation *in vitro*. For BrdU analysis, cells were plated at 10^4^ cells per well in a 96 well plate (Sigma-Aldrich) 24 hours prior to incubation with BrdU. BrdU incubation and detection was performed as per manufacturer’s instructions (Roche, Sydney, NSW, Australia). In experiments examining the cytotoxic effects of the PI3K/AKT inhibitor, BGT226, cells were treated for 48 hours with varying doses of BGT226 following which viability was determined using the Celltiter assay kit (Promega Madison, WI, USA, G3580) as described [13]. To measure basal levels of apoptosis *in vitro* Annexin V was added to a single cell suspension of Detroit 562 cells. The single cell suspension was isolated from the Detroit 562 cell line as previously described [11]. The cells were stained with Annexin V Cy 5.5 as per manufactures instructions (BD Bioscience, Sydney, NSW, Australia) and analysed using FACSCanto Diva version 2.2 Software (BD Pharminogen, Sydney, NSW, Australia).

### Generation of a stable knock down of CEACAM6 in the Detroit 562 cell line

For the generation of knock downs of CEACAM6, 2 microRNA interference (miR RNAi) sequences for CEACAM6 were made. The primers for the first miR RNAi sequence named miR CEA was, 5’ CACTGCCAAGCTCACTATTGAC 3’ for the top strand and bottom strand was 5’ GTCAATAGTGAGTGGCAGTG 3’. The other miR RNAi sequence for CEACAM6 was named miR CEA Dux, with a top strand of 5’ CCGGACAGTTCCATGTATACC 3’ and bottom stand of 5’ GGTATACATGGCTGTCCGG 3’ based on the shRNA sequence described in Duxbury et al. [16]. The pLENTI 6.1 miR RNAi sequences for miR CEA, miR CEA Dux and control (*lac Z)* were generated and transduced into to the Detroit 562 cell line as per manufacturer’s instructions (GATEWAY pLENTI cloning system, Invitrogen).

### Generation of a stable over-expression of CEACAM6 in the Detroit 562 cell line

The forward primer of 5 GGGGACAAGTTTGTACAAAAAAGCAGGCTCACCATGGGAGACCATGGGACCCCCCTCA3’ (attB1 site underlined) and reverse primer of 5’ GGGGACCACTTTGTACAAGAAAGCTGGGTGGGCTGCTATATCAGAGCCAC 3’ (attB2 site underlined) were used to generate full length CEACAM6 sequence from human epidermal keratinocytes (HEK) cDNA. The PCR conditions were as per manufactures instructions for Hifi taq (Promega). The CEACAM6 sequence was cloned into pDONR 221 (Invitrogen) using a BP reaction, then an LR reaction into pLV101G as per manufactures instructions (Invitrogen). The pLV101-Ceacam6 and pLV101 (control vector) Detroit 562 cell were generated as previously described [14].

### Tumour initiation and tumour collection

Tumour initiation studies, *in vivo* tumour treatment with the PI3K/AKT inhibitor, BGT226, and tumour sectioning were performed as previously described [11,13].

### Immunohistochemistry

Immunohistochemistry performed as previously described [11] using CEACAM6 (Biogenex, Australia), PCNA (3.2 μg/ml, Sigma-Aldrich) and Cleaved caspase 3 (0.8 μg/ml, Promega) antibodies. Control antibodies were Rabbit IgG (DAKO, Copenhagen, Denmark) and Mouse IgG (DAKO). The percentage of positive cells (PCNA and Cleaved Caspase 3) was quantified as the number of positive cells per 40x magnified field of view from a minimum of 5 to 10 randomly selected fields using NIS-Elements BR3.1 image software (Nikon, Coherent Scientific, Adelaide, SA, Australia).

### Statistical analysis

Student’s *t* test was used to assess the significance of differences between means of the different sample conditions.

## Results

### CEACAM6 expression in HNSCC

**Figure 1:**
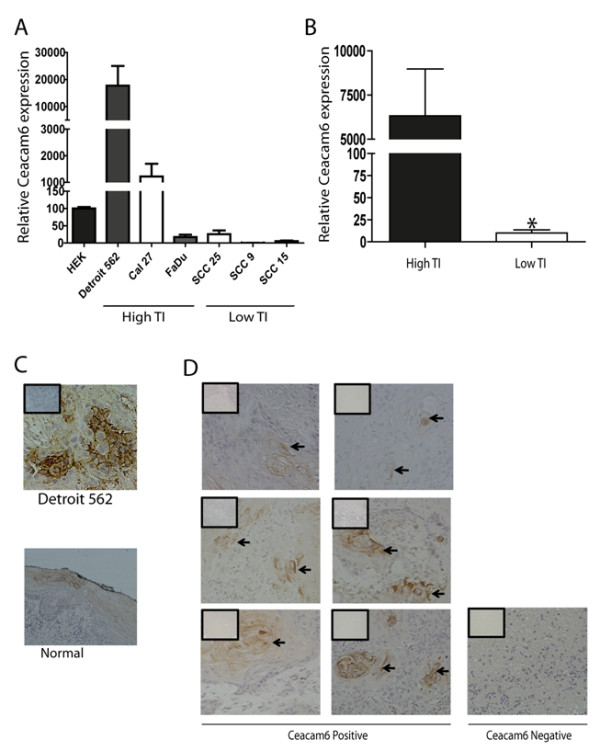
**CEACAM6 expression in HNSCC cell lines and patient tumours. A**) RtPCR analysis of CEACAM6 mRNA expression in subconfluent cultures ofHNSCC cell lines and normal HEKs (n = 4). High tumour initiating (TI) activity refers to cell lines that require less than 10^4^ cells to form a tumour and low TI activity refers to cell lines that require greater than 3x10^4^ cells to form a tumour in NOD/SCID mice. **B**) Comparison of CEACAM6 mRNA expression between high TI and Low TI cell lines (n = 4). **C**) CEACAM6 expression in a xenotransplanted tumour derived from the Detroit 562 cell line and the staining pattern for CEACAM6 in a normal oral mucosal sample. **D**) CEACAM6 expression in HNSCC patient tumours. Arrows point to areas of focal expression of CEACAM6. All images taken at 20x magnification (boxed inserts are IgG control sections). All data presented as mean +/− sem. * refers to P ≤ 0.05.

We have previously reported that CEACAM6 is over-expressed in a highly tumourigenic clonal variant of the Detroit 562 HNSCC cell line [10]. We now examine the prevalence of CEACAM6 expression in a suite of HNSCC cell lines and human HNSCC samples (Figure 1). CEACAM6 mRNA expression was 177 fold over-expressed in the Detroit 562 cell line and 12 fold over-expressed in Cal27 cell line when compared to normal human epidermal keratinocytes (HEKs, Figure 1A). We have previously reported that the Detroit 562, Cal27 and FaDu cell lines are able to form tumours in a xenotransplant model with ≤1 × 10^4^ cells whilst the SCC25, SCC9 and SCC15 cell lines are poorly tumourigenic, requiring ≥3 × 10^4^ cells to initiate a tumour [11]. Grouping the HNSCC cell lines based on tumourigenesity (highly tumourigenic ≤ 10^4^ cells or poorly tumourigenic ≥ 3 × 10^4^ cells), we were able to show an association between tumourigenesity and CEACAM6 expression (Figure 1B compare High TI *vs* Low TI). Highly tumourigenic cells had higher expression of CEACAM6 whilst poorly tumourigenic cells had relatively low levels of CEACAM6 expression (Figure 1B). However, this association is not absolute when correlating total CEACAM6 expression and tumourigenic activity. A more detailed examination of CEACAM6 expression levels by immunohistochemistry, in patient SCC samples (Figure 1D) revealed that CEACAM6 was present in 6 out of 7 patient samples (Figure 1D). All tumour samples were invasive SCC of the tongue (n = 4) or lip (n = 3). Most significantly, we found the expression of CEACAM6 to be focally overexpressed in the patient tumours which was consistent with the focal expression of CEACAM6 observed in tumours derived from the Detroit 562 parental cell line (Figure 1C) [11]. Image analysis revealed that, on average across all the tumour samples, 28% +/− 12% of the total tumour area was positive for CEACAM6 expression. However, it should be noted that the percentage area positive for CEACAM6 varied from approximately 60% down to 0% between individual tumours. Moreover, CEACAM6 positivity was often associated with keratin pearls within the tumour samples (Figure 1D). Analysis of normal human oral mucosa indicated that CEACAM6 expression is present on the plasma membranes within the suprabasal differentiated layers of the mucosa (Figure 1C). The focal expression of CEACAM6 in tumours derived from the Detroit 562 cell line was consistent with our earlier study reporting that clonal variants existed within the parental Detroit 562 cell line that could be discriminated based on variant-specific transcriptomic signatures [11]. These findings highlight 2 important observations. Firstly, the majority of HNSCC have foci of CEACAM6 overexpression. Secondly, examining global expression of CEACAM6, at a tissue level, is not a good indicator of the presence or abundance of CEACAM6^+ve^ foci/clonal variants within cell cultures or tumours. The concept of intratumoural heterogeneity has recently been validated by single cell sequencing techniques in patient tumours and has significant implications for tumour progression and drug resistance [17].

### The role of CEACAM6 in HNSCC tumourigenesity

**Figure 2:**
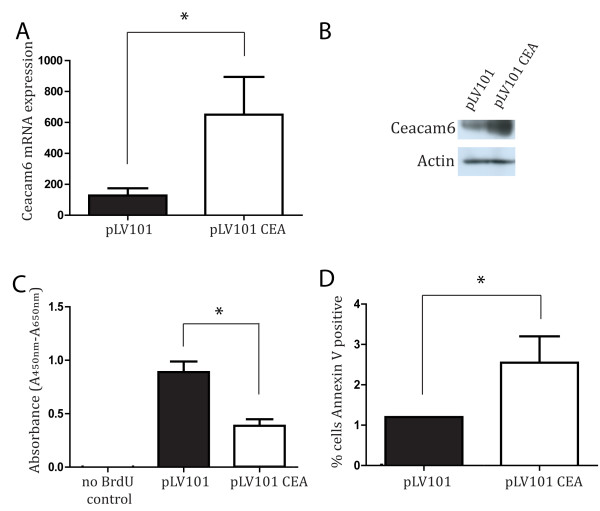
**Impact of CEACAM6 over-expression on proliferation and apoptosis in the Detroit 562 cell line. A**) Rt-PCR analysis of CEACAM6 mRNA expression in pLV101 control and pLV101 CEA transduced Detroit 562 cells. **B**) CEACAM6 protein expression in control and pLV101 CEA transduced Detroit 562 cells. β-actin is provided as a reference for loading equivalence. **C**) BrdU incorporation in control (PLV101) and pLV101 CEA transduced Detroit 562 cells (n = 6). BrdU is reported as Absorbance Units/well (A_450nm_-A_560nm_). **D**) Percentage of total cells that express Annexin V in control (PLV101) and pLV101 CEA transduced Detroit 562 cells. Data presented as mean +/− sem of at least 3 independent experiments performed in triplicate. * P ≤ 0.05.

**Figure 3:**
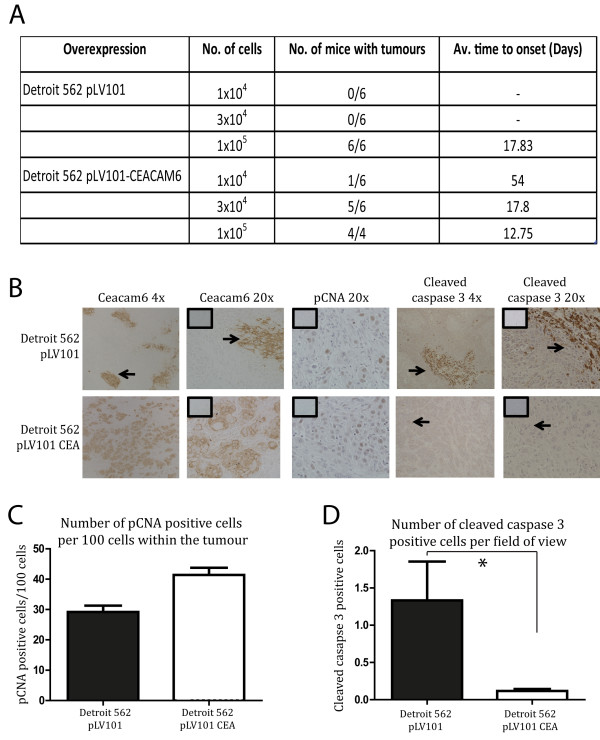
**Effect of CEACAM6 overexpression on tumour initiation. A**) Varying numbers of control (PLV101 control) or CEACAM6 overexpressing (PLV101 CEA) Detroit cells were assayed for their ability to initiate tumours in NOD/SCID mice. Mice (6/group) were monitored over a 12 week period and the incidence of tumours and the time till tumours were palpable is recorded. **B**) CEACAM6, PCNA and cleaved Caspase 3 expression was determined immunohistochemically at 4x and 20x magnification in xenotransplant tumours derived from pLV101 control and pLV101 CEACAM6 over-expressing cells (insets are IgG control sections). **C**, **D**) PCNA positivity in the CEACAM6 over-expressing and control tumours was estimated as the number of positive cells per 100 cells in a field of view by NIS-Elements BR3.1 imaging software. Quantification of cleaved caspase 3 expression (**D**) was estimated as number of positive cells per field of view by NIS-Elements BR3.1 imaging software. All data expressed as mean +/− sem. * P ≤ 0.05.

CEACAM6 is i) overexpressed focally in SCC, ii) overexpressed in SCC cell lines and iii) CEACAM6 expression level correlates with tumour initiating activity. Therefore, we used the Detroit 562 cell line to examine the contribution of CEACAM6 to tumour initiating activity and/or tumour growth. CEACAM6 overexpression was achieved using a lenti-viral over-expression vector (Figure 2A, B). To determine whether the over-expression of CEACAM6 was able to modulate proliferation and cell death, BrdU and Annexin V assays were performed *in vitro* (Figure 2C, D). The BrdU assay for proliferation indicated a 5-fold increase in CEACAM6 expression was associated with a 50% reduction in proliferation in the Detroit 562 cell line *in vitro* (Figure 2C). In contrast, CEACAM6 overexpression significantly enhanced Annexin V positivity *in vitro* (Figure 2D). Next, we examined the effect of overexpressing CEACAM6 in Detroit 562 cells on tumour initiation and growth *in vivo* in our xenotransplant model. CEACAM6 overexpressing SCC cells (Detroit 562 pLV101-CEACAM6) were able to initiate tumours with 1 × 10^4^ cells whereas vector-infected control cells (Detroit 562 pLV101) required 1 × 10^5^ cells to initiate a tumour (Figure 3A). Immunohistochemical staining confirmed that overexpression of CEACAM6 persisted *in vivo* to the termination of the study (Figure 3B). Finally, we found that overexpression of CEACAM6 resulted in a modest increase in the expression of the proliferation marker, PCNA, when compared to control tumours (Figure 3C). Significantly, overexpression of CEACAM6 in Detroit 562 cells was accompanied by a profound and significant decrease in the apoptotic index of tumour cells *in vivo* compared to control tumours (Figure 3D). These data indicate the enhanced tumour growth observed in the CEACAM6 over-expressing cells was predominantly attributable to a decrease in caspase 3-dependent cell death *in vivo*. These effects were not observed *in vitro* and suggest that CEACAM6-mediated alterations in tumour proliferation and apoptosis are regulated by factors specific for the microenvironment in which the tumours reside *in vivo*. Differences in *in vitro* and *in vivo* apoptotic responses are not unexpected. We have previously reported that agents such as histone deacetylase inhibitors exhibit substantial cytotoxic effects on SCC cells *in vitro* yet fail to induce cytotoxicity against SCC cells in xenotransplant models or human subjects [13,18]. Moreover, a recent study reported that stromal elements are able to modify tumour cell sensitivity to cytotoxic drugs [19].

**Figure 4:**
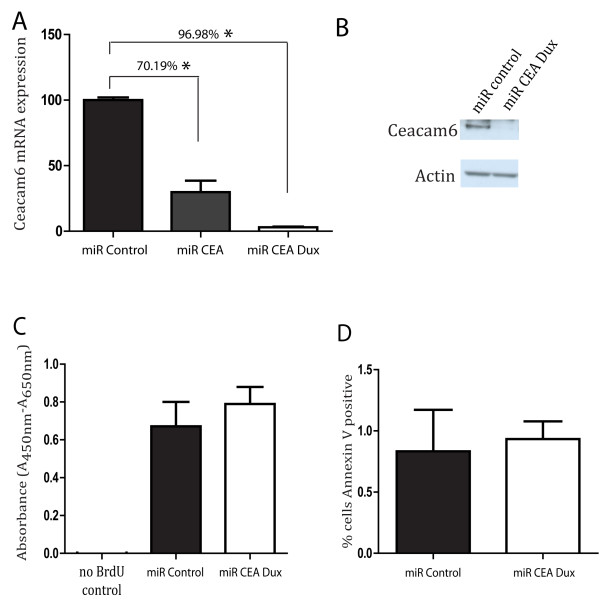
**Knock down of CEACAM6 in the Detroit 562 cell line. A**) The mRNA expression of CEACAM6 in control miR RNAi and 2 miR RNAi knock down sequences (miR CEA and miR CEA Dux) was quantified by rtPCR analysis. **B**) The knock down of CEACAM6 using miR CEA Dux was confirmed at the protein level using western blot analysis. **C**) BrdU incorporation in control (miR Control) and knock down detroit cells (miR CEA Dux) is shown (n = 6). BrdU is reported as Absorbance Units/well (A_450nm_-A_560nm_). **D**) Percentage of total cells that express Annexin V positivity in control (miR Control) and knockdown Detroit cells (miR CEA Dux) is shown. Data presented as mean +/− sem of at least 3 independent experiments performed in triplicate. * P ≤ 0.05.

**Figure 5:**
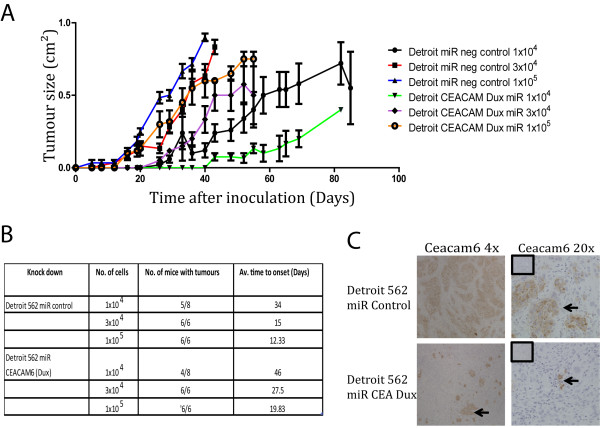
**Tumour initiation studies of CEACAM6 knock down. A**, **B**). Tumour initiation study was performed with the CEACAM6 knock down (miR CEA Dux) and control (miR Control) cells as described in Figure 3 (6 mice/group). **C**) Expression of CEACAM6 in the CEACAM6 knock down and control xenotransplant tumours at 4x and 20x magnification (inserts are of IgG control sections).

Next, we investigated whether reducing CEACAM6 expression would also be able to modulate tumour initiation and growth in the Detroit 562 cell line. Efficiency of knock down of CEACAM6 was achieved using 2 miR RNAi sequences, miR CEA and miR CEA Dux, and was measured by rt PCR (Figure 4A). CEA Dux sequence had the greatest knock down of the 2 sequences, with 96.98% knock down at the mRNA level. Using the CEA Dux sequence, the knock down of CEACAM6 was confirmed at the protein level (Figure 4B). BrdU and Annexin V assay analysis indicated that knock down of CEACAM6 in the Detroit 562 cells had no significant effect on the proliferative potential or basal levels of cell death compared to control cells (Figure 4C and D). This would suggest that the modest effects of overexpression of CEACAM6 on proliferation and apoptosis observed in an *in vitro* setting may be an artefact of overexpression. Next, we examined the ability of CEACAM6 Dux (miR CEA Dux) transduced or control-transduced (miR Control) cells to establish tumours in a xenotransplant model (Figure 5A, B). CEACAM6 knockdown cells took longer to establish and grow than control cells (Figure 5A, B). Immunohistochemistry confirmed that knock down of Ceacam6 persisted to the termination of the study in xenotransplanted tumours (Figure 5C). These data indicate that CEACAM6 expression was reduced, but not completely ablated, in the CEACAM6-knock down tumours when compared to control tumours. Combined, the overexpression and knockdown studies show that CEACAM6 can enhance the tumourogenesity of HNSCC cells. Moreover, we show that CEACAM6 overexpression enhances tumourogenesity by inhibiting apoptosis.

**Figure 6:**
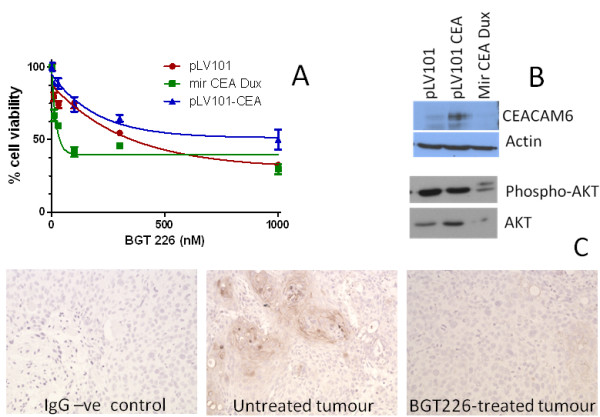
**CEACAM6 expression in tumours treated with a PI3K/AKT inhibitor. A**) Subconfluent cultures of Detroit 562 cells transduced with vector (pLV101), knockdown construct (mir CEA Dux) or overexpression vector (pLV101-CEA) were exposed to varying concentrations of BGT226 for 48 hours after which viability was assessed. Data presented as mean ± sem from 2 experiments performed in triplicate. **B**) Protein was harvested from untreated cells used in (A) and CEACAM6, AKT or phospho-S437 AKT protein expression estimated by western blot. β-actin expression is presented to confirm loading equivalence. **C**) 10^6^ cells used in (A) were injected into NOD/SCID mice and when tumours reached 0.4 cm^3^ the mice were treated with daily doses of vehicle or BGT226 as described elsewhere [13]. Mice were sacrificed when tumours reached 1 cm^3^ and tumours removed, fixed and 5 μm sections stained for CEACAM6 expression. Note mice treated with BGT226 displayed significant reductions in tumour growth and time to sacrifice was significantly extended in these mice [13]. An IgG negative control is shown on the left. Six mice per group were used and a representative section from one mouse is shown. 20X magnification.

We have shown that CEACAM6 can increase tumour initiating activity and inhibit apoptosis (Figures 3 and 4). Thus, we were interested in whether the antiapoptotic effects of CEACAM6 could extend to the suppression of cytotoxic activity of a PI3K/AKT/mTOR inhibitor, BGT226 [13]. Human SCC frequently harbor defects in survival pathways such as the PTEN/PI3K/AKT/mTOR pathway which can attenuate responses to chemotherapeutics [see 15]. Moreover, it has been previously reported that CEACAM6 can inhibit cytotoxicity induced by a conventional chemotherapeutic, gemcitabine, in pancreatic cancer cells [9]. Anticancer treatments are increasingly relying on the use of targeted therapies [17] and we have previously shown that targeting the PI3K/AKT/mTOR pathways in HNSCC shows considerable anticancer activity in xenotransplant models of HNSCC [13]. We compared the sensitivity of Detroit 562 cells (PLV101) (Figure 6) to the PI3K/AKT inhibitor, BGT226, with the sensitivity of Detroit 562 cells in which CEACAM6 is overexpressed (PLV101-CEA) (6A) or knocked down by stable expression of an shRNA (mir CEA-Dux) (Figure 6A). Figure 6 shows that inhibition of CEACAM6 enhances sensitivity of SCC cells to BGT226 (Ec50 shifts from 230 nM in PLV101 to 14.3 nM in mir CEA-Dux cells). Overexpression of CEACAM6 reduces the sensitivity (Ec50 = 138 nM in PLV101-CEA) and maximal response to BGT226 (Figure 6) (Max response = 70% kill in PLV101 and 50% in PLV101-CEA). Moreover, we show that overexpression of CEACAM6 causes an induction of AKT whilst knockdown of CEACAM6 causes a reduction in total and phospho-S473 AKT (Figure 6B). These data indicate that CEACAM6 is a modulator of the constitutive PI3K/AKT survival pathway in SCC cells and is able to modulate the cytotoxic response to pharmacological inhibitors of the PI3K/AKT pathway. Finally, we had previously reported that SCC cells when grown, in a xenotransplant model, display initial transient sensitivity to BGT226 followed by the expansion of BGT226-resistant cells [13]. We now report that 4 weeks of daily treatment with BGT226 of mice bearing tumours derived from Detroit 562 cells selectively ablates CEACAM6-positive foci in the tumours (Figure 6B).

## Discussion

In this study we report, for the first time, on the role of CEACAM6 in HNSCC. Previous work with keratinocytes and keratinocyte-derived SCC cells has shown that CEACAM6 is selectively expressed in differentiated keratinocytes [2] and is highly expressed in a tumourigenic clonal variant of the Detroit 562 HNSCC cell line [10]. In addition, other workers have reported that i) CEACAM6 overexpression occurs in variety of epithelial malignancies [5-7], ii) that CEACAM6 overexpression is associated with increased metastases, proliferation and the suppression of annoikis [7-9], iii) that CEACAM6 overexpression induces a *src*-dependent increase in AKT activity that suppresses gemcitabine sensitivity in pancreatic cancer cells [9] and finally, iv) a transgenic model of CEA-overexpression suggests CEACAM6 overexpression can contribute to the development of colonic dysplasia [10]. We now extend these findings and report that CEACAM6 is focally overexpressed in a large fraction of human HNSCCs *in situ.* The heterogeneous pattern of CEACAM6 overexpression is also evident in established HNSCC cell lines *in vitro* and *in vivo*. Moreover, we show that over-expression of CEACAM6 increases tumour growth and tumour initiating activity by suppressing PI3K/AKT-dependent apoptosis of HNSCC in a xenotransplant model of HNSCC. Finally, we show that foci of CEACAM6 expressing cells are selectively ablated by treatment of xenotransplant tumours with pharmacological inhibitors of PI3K/AKT *in vivo*.

A novel finding in the present study is the observation that CEACAM6 is focally overexpressed in the majority of HNSCCs examined. Whilst the sample size examined was small it highlights an important issue that has important biological and clinical implications. Specifically, intratumoural heterogeneity is a major contributor to the emergence of drug resistance and tumour recurrence [17]. Consistent with this, our data suggest that focal overexpression of CEACAM6 is indicative of sensitivity of human HNSCC to selective cytotoxic drugs. In this regard two observations relating to CEACAM6 are relevant. Firstly, knockdown or overexpression of CEACAM6 resulted in a decrease and increase in tumourigenic activity in SCC cells *in vivo* respectively. Secondly, CEACAM6 has been shown to modulate the cytotoxic effects of conventional chemotherapeutics such as gemcitabine in pancreatic cancer cell lines [9] and in the present study we showed that CEACAM6 could mediate sensitivity to new targeted agents such as the PI3K inhibitor, BGT226. It is noteworthy that the modulation of gemcitabine sensitivity is also mediated *via* a *src* and PI3K/AKT-dependent pathway [9]. These data indicate that whilst CEACAM6 may invoke pro-survival responses in cancer cells by activating the PI3K/AKT pathway this same pathway could be selectively targeted by specific cytotoxic drugs. Thus, the presence of CEACAM6^+ve^ foci would be predicted to bestow selective sensitivity against certain chemotherapeutic treatments (eg: gemcitabine or PI3K inhibitors). Proof of principle for this hypothesis is shown by the reduction in phospho-S437 AKT induced by knockdown of CEACAM6 and the loss of CEACAM6^+ve^ foci in tumours treated with cytotoxic doses of PI3K inhibitors. Thus, CEACAM6 could be used to predict PI3K inhibitor sensitivity. Moreover, the observation that CEACAM6 expression correlates with metastatic potential [8,20-22] would suggest that, in chemotherapy-naive tumours, the presence of CEACAM6^+ve^ foci could serve as a prognostic marker of poor outcome and in this instance targeting CEACAM6/PI3K/AKT pathways could be exploited therapeutically. Supporting this, is a recent study, by Blumenthal et al. [20], demonstrating that the addition of antibodies that inhibited the binding of CEACAM6^+ve^ breast cancer cells to endothelial cells reduced tumour cell invasion [20]. Finally, intratumoural heterogeneity can arise through a number of mechanisms such as the evolution of variant cells from a common clonal precursor, micro-environmental influences, stochastic processes or tissue/cell plasticity [17]. The present study suggests that the focal pattern of CEACAM6 expression, in tumours, is derived from a specific clonal progenitor within the tumour rather than being transiently induced by the local environment. This is based on the observation that CEACAM6^+ve^ and ^–ve^ cells persist in long term tissue culture models, consistent with an heritable mechanism (eg: genetic or epigenetic).

Whilst CEACAM6 clearly has the capacity to contribute to drug resistance and tumour recurrence it is clear that other factors also contribute to drug resistance and tumour recurrence. This is supported by our observation that targeted inhibition of the CEACAM6/PI3K/AKT pathway in SCC cells induced killing of 50% of the total HNSCC cells. Similarly, we have identified clonal variants of HNSCC cells that express very low levels of CEACAM6 yet still retain tumourigenic potential [11]. Moreover, we show that the knockdown of CEACAM6 results in a decrease, but not an ablation, of tumour initiating activity or tumour growth. Thus, CEACAM6 likely represents one factor, of many, that can modulate tumour growth and tumour initiating activity. This is entirely consistent with the emerging importance of intratumoural heterogeneity [17]. We previously reported that HNSCC display intratumoural heterogeneity that was reflected in histomorphologically and transcriptomically distinct clonal variants [11,14]. We showed that clonal variants of HNSCC cells could persist *in vitro* in established cell lines and displayed significant differences in tumour initiating activity and drug resistance [11,13,14]. Several groups have now definitively shown, by single cell sequencing, that tumours comprise multiple genetically distinct clonal populations [23-27]. Emerging, clinical and molecular data unequivocally show that the presence of intratumoural heterogeneity, exemplified by focal CEACAM6 overexpression in HNSCC cells, is a major contributor to tumour drug responses and patient outcomes [17].

Earlier work by Duxbury [28], suggests that the major contribution of CEACAM6 to tumour growth and tumour initiating activity is mediated *via* suppression of anoikis. Anoikis is a form of apoptosis induced by loss of cell-cell/EMC contact. Thus, anoikis may be more relevant to a 3 dimensional tumour environment rather than an *in vitro* cell monolayer system. [29]. Supporting this, we found that the *in vivo* effects of CEACAM6 over-expression/knockdown were not reflected by the *in vitro* effects of CEACAM6. For instance, CEACAM6 over-expression/knockdown had modest and inconsistent effects on apoptotic rates *in vitro.* However, over-expression of CEACAM6 significantly reduced caspase-3 dependent apoptosis of HNSCC cells in a xenotransplant model. Anti-apoptotic activity is commonly viewed as tumour promoting and hence the anti-apoptotic activity of CEACAM6 would suggest it has tumour promoting (oncogenic) activity [30]. CEACAM6-mediated inhibition of apoptosis *in vivo* therefore contributes in part, or wholly, to the ability of HNSCC cells to initiate a tumour in a xenotransplant model of HNSCC. In addition, CEACAM6 over-expression also contributes in part, or wholly, to the increased tumour growth in a xenotransplant model of HNSCC. Based on these findings, it is reasonable to speculate that focal patches of CEACAM6 expressing cells within HNSCC may reflect the presence of a subpopulation of cells with a greater potential for recurrence/metastasis than CEACAM6^-ve^ subpopulations of HNSCC cells.

## Conclusions

In conclusion, our study shows that CEACAM6 is focally overexpressed in a large fraction of human HNSCCs *in situ* and contributes to tumour growth and tumour initiating activity. The effect of CEACAM6 on tumour growth and initiation is mediated *via* suppression of PI3K/AKT-dependent apoptosis of HNSCC in a xenotransplant model of HNSCC. Finally, our studies show that CEACAM6^+ve^ tumours, or tumour foci, are selectively sensitive to treatment with pharmacological inhibitors of PI3K/AKT *in vivo*.

This work was supported by a PhD scholarship awarded to SC by the Garnett Passe & Rodney Williams Memorial Foundation. NS is supported by a senior research fellowship awarded by the Cancer Council Queensland. This work was also supported by a research grant awarded to NS (#455929, #569689) from the Australian NHMRC: Cancer Council QLD, #631479 and a practitioner fellowship to AG from the Cancer Collaborative Group.

## Competing interests

The authors declare that they have no competing interests.

## Authors’ contributions

SC drafted the manuscript and performed *in vitro* growth and death assays. LMDL performed the animal experiments. MHR, ET and LEM performed immunohistochemistry and cloning experiments. AC and OG performed the drug toxicity studies. AG and NS supervised the project and contributed to the draft of manuscript. All authors read and approved the final manuscript.
